# Fluvoxamine for blonanserin-associated akathisia in patients with schizophrenia: report of five cases

**DOI:** 10.1186/1744-859X-9-17

**Published:** 2010-04-24

**Authors:** Tsutomu Furuse, Kenji Hashimoto

**Affiliations:** 1 Department of Psychiatry, Asahikawa Red Cross Hospital, Asahikawa, Japan; 2 Division of Clinical Neuroscience, Chiba University Center for Forensic Mental Health, Chiba, Japan

## Abstract

**Background:**

Atypical antipsychotic drugs have been reported to cause fewer incidences of extrapyramidal side effects (EPS) than typical antipsychotic drugs, but adverse events such as akathisia have been observed even with atypical antipsychotic drugs. Although understanding of the pathophysiology of akathisia remains limited, it seems that a complex interaction of several neurotransmitter systems plays a role in its pathophysiology. The endoplasmic reticulum protein sigma-1 receptors have been shown to regulate a number of neurotransmitter systems in the brain.

**Methods:**

We report on five cases in which monotherapy of the selective serotonin reuptake inhibitor and sigma-1 receptor agonist fluvoxamine was effective in ameliorating the akathisia of patients with schizophrenia treated with the new atypical antipsychotic drug blonanserin.

**Results:**

The global score on the Barnes Akathisia Scale in five patients with schizophrenia treated with blonanserin rapidly decreased after fluvoxamine treatment.

**Conclusion:**

Doctors should consider that fluvoxamine may be an alternative approach in treating akathisia associated with atypical antipsychotic drugs.

## Background

Atypical antipsychotic drugs have been reported to cause a fewer incidences of extrapyramidal side effects (EPS) than typical antipsychotic drugs, but adverse events such as akathisia have been observed even with atypical antipsychotic drugs. Akathisia is one of the common and distressing EPS of antipsychotic drugs [1,2]. The development of akathisia can adversely affect patients' adherence to medication, and, as a consequence, have a negative impact on long-term treatment outcomes in patients with schizophrenia [3,4]. Although therapeutic drugs (for example, β-adrenergic blockers, benzodiazepines, and anticholinergic drugs) have been used in the treatment of akathisia, they show only a moderate efficacy, and a substantial proportion of patients fail to respond to treatment. In contrast, understanding of the pathophysiology of akathisia remains limited. Given the clinical profile of akathisia, it seems that a complex interaction of several neurotransmitter systems (for example, dopamine, acetylcholine, norepinephrine, serotonin, γ-aminobutyric acid (GABA), and neuropeptides) underlies its complex pathophysiology [1,2].

The endoplasmic reticulum protein sigma-1 receptors play a key role in Ca^2+ ^signaling and cell survival, and have been shown to regulate a number of neurotransmitter systems in the central nervous system [5-8]. A recent study identified the sigma-1 receptors as possessing innate biological activity as a molecular chaperone, activity that can be activated/inactivated by synthetic compounds that bind to sigma-1 receptors [9,10]. Furthermore, sigma-1 receptors play important roles in Ca^2+ ^signaling and bioenergetics within the cell [8-10]. The selective serotonin reuptake inhibitor fluvoxamine is a very potent agonist at sigma-1 receptors [11,12]. A study using a selective sigma-1 receptor agonist [^11^C]SA4503 and positron emission tomography demonstrated that fluvoxamine binds to sigma-1 receptors in the living human brain at therapeutic doses, suggesting that sigma-1 receptors might play a role in the mechanism of action of fluvoxamine [13]. Given the important role of sigma-1 receptors in the regulation of neurotransmitter systems, we hypothesized that fluvoxamine may be effective in the treatment of akathisia associated with antipsychotic treatment. Very recently, we reported on cases in which fluvoxamine was effective in treating aripiprazole-induced akathisia in patients with schizophrenia, suggesting that fluvoxamine would also be a potential therapeutic drug for antipsychotic-induced akathisia [14].

Blonanserin (AD-5423; trade name Lonasen) is a new atypical antipsychotic drug that has the properties of both a serotonin 5-HT_2A _and a dopamine D_2 _receptor antagonist [15], and this drug has been used in Japan and South Korea. The affinity of this drug at dopamine D_2 _receptors is higher than that at serotonin 5-HT_2A _receptors [15]. A randomized, double-blind, placebo-controlled and haloperidol-controlled international multicenter study demonstrated that blonanserin was effective in the treatment of acute schizophrenia, and that it had greater efficacy in negative symptoms compared with placebo and haloperidol [16]. In addition, blonanserin was well tolerated and its safety profile compared favorable with haloperidol, particularly with respect to prolactin elevation and EPS frequency [16]. We have experienced that treatment with blonanserin might cause akathisia in some patients with schizophrenia, although the data on blonanserin-associated akathisia have not yet been published. Here we report five cases where fluvoxamine was effective in treating blonanserin-associated akathisia in patients with schizophrenia.

## Case reports

Table 1 shows the characteristics of five patients with blonanserin-associated akathisia.

**Table 1:** Characteristics of five schizophrenic patients with blonanserin-associated akathisia.

Case	Gender (F/M)	Age (years)	Dose of blonanserin	Barnes AKS score before treatment	Barnes AKS score after treatment
1	M	40	8-16 mg	3	0

2	F	48	8-24 mg	3	0

3	M	63	8 mg	2	0

4	M	36	8 mg	3	0

5	M	42	16-24 mg	3	0

Barnes AKS = Barnes Akathisia Scale.

### Case 1

The patient was a 40-year-old man who met the *Diagnostic and Statistical Manual of Mental Disorders, fourth edition *(DSM-IV) criteria for schizophrenia. Onset of schizophrenia occurred in his early twenties. The typical antipsychotic drug haloperidol was administered for some time. After he stopped the medication (haloperidol 2 mg) because of tremor, he jumped from a second floor window due to delusions and hallucinations. He was then admitted to the hospital's emergency medical center with a right calcaneal fracture. His right leg joint was fixed in a plastic cast, and he was admitted. Treatment with blonanserin (8 mg) and flunitrazepam (2 mg, at night) was initiated for auditory hallucination. At 1 week later, the blonanserin was increased to 16 mg because his persecutory delusions and auditory hallucinations persisted. After the increase in dose (16 mg), the patient complained of leg restlessness. His global score on the Barnes Akathisia Scale [17] was 3 ('moderate akathisia'). Substantial relief of akathisia was noted on the next day of fluvoxamine (50 mg) treatment, at which point his global score on the Barnes Akathisia Scale was 0. At 1 week later, the dose of blonanserin was increased to 24 mg since he still had psychotic symptoms. Fluvoxamine (50 mg) continued to be administered. He had no recurrence of the akathisia. After 1 month, he was discharged home because his psychotic symptoms were improved.

### Case 2

The patient was a 48-year-old woman who met the DSM-IV criteria for schizophrenia. The onset of schizophrenia occurred in her late twenties, and 10 years previously she had been admitted to a hospital emergency medical center with delusions and hallucinations. She was treated for this previous episode with haloperidol (6 mg), but she had stopped the medication due to EPS. She was admitted to the hospital emergency medical center with a recurrence of psychosis, including delusions and hallucinations. Treatment with blonanserin (8 mg), flunitrazepam (2 mg, at night), and levomepromazine (50 mg) was initiated. At 3 days later, the patient complained of leg restlessness. Her global score on the Barnes Akathisia Scale [17] was 3. Substantial relief of akathisia was noted on the next day of fluvoxamine (50 mg) treatment, at which point her global score on the Barnes Akathisia Scale was 0. The dose of blonanserin was increased to 24 mg due to an abnormal experience. Fluvoxamine (50 mg) continued to be administered. After 3 weeks, she was discharged home because she had no recurrence of the akathisia.

### Case 3

The patient was a 63-year-old man who met the DSM-IV criteria for schizophrenia. The onset of schizophrenia occurred in his early twenties. He had been treated with olanzapine (10 mg) for the last 4 years, but he had a tendency to stop the medication due to appetite and body weight. He was admitted to the hospital due to delusions and hallucinations at his older brother's funeral. Treatment with blonanserin (8 mg) and etizolam (1 mg, at night) was initiated for auditory hallucinations and delusions. At 2 weeks later, the patient complained of leg restlessness. His global score on the Barnes Akathisia Scale [17] was 2. Substantial relief of akathisia was noted after 14 days of fluvoxamine (50 mg) treatment. His global score on the Barnes Akathisia Scale was 0.

### Case 4

The patient was a 36-year-old man who met the DSM-IV criteria for schizophrenia. The onset of schizophrenia occurred in his early twenties. He was admitted to the hospital's emergency medical center with a cut to the right of the neck from an unsuccessful suicide attempt. Treatment with blonanserin (8 mg) and flunitrazepam (2 mg, at night) was initiated for auditory hallucinations. At 2 days later, the patient complained of leg restlessness. His global score on the Barnes Akathisia Scale [17] was 3. Substantial relief of akathisia was noted on the next day of fluvoxamine (50 mg) treatment. His global score on the Barnes Akathisia Scale was 0. Blonanserin (8 mg), fluvoxamine (50 mg), and flunitrazapam (2 mg) continued to be administered. After 2 weeks, he was discharged home because he had recovered.

### Case 5

The patient was a 42-year-old man who met the DSM-IV criteria for schizophrenia. The onset of schizophrenia occurred in his early twenties. He had been treated with haloperidol (18 mg), chlorpromazine (150 mg), biperiden (6 mg), and haloperidol decanoate (100 mg) for some time. He tended to drink a lot of water due to mouth dryness. He was admitted to the hospital's emergency medical center because he had fallen at home. He was diagnosed with low sodium blood syndrome due to water intoxication, and he was treated with intravenous nutrition. After recovery, he was treated with blonanserin (16 mg) and flunitrazapam (2 mg, at night). At 2 days later, the patient complained of leg restlessness after the increase in blonanserin (24 mg). His global score on the Barnes Akathisia Scale [17] was 3. Substantial relief of akathisia was noted on the next day of fluvoxamine (50 mg) treatment, at which point his global score on the Barnes Akathisia Scale was 0. After 1 week, he was discharged home because she had no psychotic symptoms.

## Discussion

To our knowledge, this is the first report demonstrating that fluvoxamine is rapidly effective in the treatment of blonanserin-induced akathisia in patients with schizophrenia. Very recently, we reported that fluvoxamine is also effective in the treatment of aripiprazole-induced akathisia in patients with schizophrenia [14]. Nevertheless, a further randomized double-blind, placebo-controlled study of fluvoxamine will be needed to confirm its efficacy for the treatment of akathisia. From the present study, it is unclear whether sigma-1 receptor agonism appears to be irrelevant to the rapid antiakathitic action of fluvoxamine. In order to confirm the role of sigma-1 receptors in the treatment of akathisia, a randomized double-blind, placebo-controlled study of the selective sigma-1 receptor agonist (for example, cutamesine (SA4503)) in patients with antipsychotic-induced akathisia will be necessary.

Akathisia is a neurological side effect of antipsychotic medications, which are used to treat various psychiatric disorders such as schizophrenia and bipolar disorders [1,2,4]. It seems that akathisia is simply a dopamine D_2 _receptor blockade [1], although the precise mechanisms underlying antipsychotic drug-induced akathisia are currently unclear. A number of neurotransmitter systems play a role in the complex pathophysiology of akathisia [1,2]. At present, it is unclear whether sigma-1 receptor agonism is involved in the mechanism of the rapid antiakathitic action of fluvoxamine. Considering the important role of sigma-1 receptors in the regulation of a number of neurotransmitter systems [5-8], it is likely that indirect modulation of several neurotransmitter systems by sigma-1 receptor agonist may be involved in the mechanisms of this drug, although further detailed study will be necessary [14].

## Conclusions

These five cases suggest that fluvoxamine may serve as an alternative option in the treatment of antipsychotic-induced akathisia in patients with schizophrenia. Further detailed randomized, double-blind studies of the selective sigma-1 receptor agonist using larger samples should be performed to clarify the role of sigma-1 receptors in the efficacy of fluvoxamine for akathisia.

## Consent

Written informed consent was obtained from all patients in this case report after we explained the fact that fluvoxamine use for akathisia is off label.

## Competing interests

The authors declare that they have no competing interests.

## Authors' contributions

TF contributed to the clinical and rating evaluations during the follow-up periods. KH conceived of the study and participated in its study and coordination. All authors read and approved the final manuscript.
